# Multiscale Feature Fusion for Skin Lesion Classification

**DOI:** 10.1155/2023/5146543

**Published:** 2023-01-05

**Authors:** Gang Wang, Pu Yan, Qingwei Tang, Lijuan Yang, Jie Chen

**Affiliations:** ^1^College of Electronic and Information Engineering, Anhui Jianzhu University, Hefei 230000, China; ^2^Anhui International Joint Research Center for Ancient Architecture Intellisencing and Multi-Dimensional Modeling, Anhui Jianzhu University, Hefei 230000, China; ^3^Anhui Provincial Key Laboratory of Intelligent Building and Building Energy Conservation, Anhui Jianzhu University, Hefei 230000, China

## Abstract

Skin cancer has a high mortality rate, and early detection can greatly reduce patient mortality. Convolutional neural network (CNN) has been widely applied in the field of computer-aided diagnosis. To improve the ability of convolutional neural networks to accurately classify skin lesions, we propose a multiscale feature fusion model for skin lesion classification. We use a two-stream network, which are a densely connected network (DenseNet-121) and improved visual geometry group network (VGG-16). In the feature fusion module, we construct multireceptive fields to obtain multiscale pathological information and use generalized mean pooling (GeM pooling) to reduce the spatial dimensionality of lesion features. Finally, we built and tested a system with the developed skin lesion classification model. The experiments were performed on the dataset ISIC2018, which can achieve a good classification performance with a test accuracy of 91.24% and macroaverages of 95%.

## 1. Introduction

**Figure 1:**
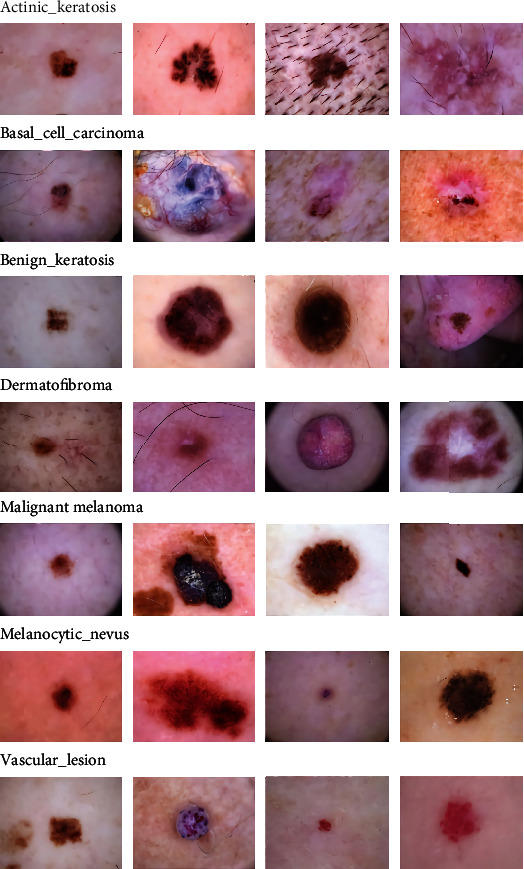
Seven classes of pigmented skin lesions.

The skin epidermis consists of three kinds of cells: basal, squamous, and melanocyte [1]. Pigmented skin diseases [2] are formed because of abnormalities in melanin and melanocytes. The deadliest skin disease is melanoma, a highly malignant tumor derived from melanocytes. Due to changes in skin color and inconspicuous early lesions, it is very difficult to classify them. However, it can be cured if detected and treated in time, so the accurate classification of pigmented skin diseases is particularly important for later treatment [3]. Clinically, it is a common method to use modern skin imaging technology and expert experience for treatment and diagnosis [4]. However, the subjective factors of experts are not fully authoritative, and imaging analysis is time-consuming and laborious, so it is difficult to automatically classify malignant skin diseases from dermoscopic images. With the development of computer image processing technology, computer-aided design (CAD) system [5] can realize automatic classification based on skin lesion images. However, pigmented skin diseases have high interclass similarity and intraclass variations, and the pathological characteristics of early and late stages are completely different. Benign and malignant diseases have little difference in early symptoms, but when the lesions are formed, the appearance is very different, for example, melanoma (malignant) and melanoma nevus (benign). Both diseases are tumors derived from melanocytes, and their clinical manifestations are the same, which makes classifying them difficult.  Figure 1 shows the early symptoms and later manifestations of the seven skin lesions. The first column is the early stage of seven skin lesions, and the others are random symptoms of skin lesions. We can see that the color, boundary size, and abnormal appearance of lesions in the early stage are very similar, while the appearance of late stage is very different. Therefore, it is impossible to infer with the naked eye whether the early skin lesions will become malignant or benign. It makes it more difficult to classify them.

Accurate skin lesion classification remains a challenge due to four factors. Firstly, image classification technology based on CNN network requires a large number of training datasets. However, due to the scarcity and imbalance of medical datasets, it is difficult to collect a large number of skin lesion images. Thus, we use six data augmentation operations to expand and balance the ISIC2018 dataset. Secondly, local pathological information is an important judgment to improve the classification accuracy. To fully mine the local pathological information of skin lesion images, we introduce a residual structure to simplify the learning process and allow the network to learn the local and global features of skin lesion images. Thirdly, feature fusion is an important means to improve classification performance. Since the pathological features obtained from a single network are limited and feature correlation affects the classification performance, we use a two-stream network to eliminate the redundant information brought by the correlation between different feature sets. Finally, a single receptive field cannot obtain more comprehensive lesion information. Therefore, we use multireceptive fields (small convolution kernel and large convolution kernel) to complement each other and make the network get more pathological regions. Overall, the contributions of this paper are as follows:

- We fuse the residual structure for the traditional VGG-16 network model to reduce the risk of gradient disappearance in the network
- We use a two-stream network model (DenseNet-121 and VGG-16) for feature fusion, which can combine the advantages of a single network. And then, we use the multireceptive field module to obtain multiscale pathological information
- A skin lesion classification system is designed, which provides accurate diagnostic information for experts or patients

## 2. Related Works

### 2.1. DCNN-Based Skin Lesion Classification

To apply deep convolutional neural network (DCNN) to skin lesion classification [6, 7], experts and scholars have innovated many classical network structures. He et al. [8] introduced a deep residual learning framework to solve the degradation problem, making it possible to build very deep networks, such as AlexNet [9], VGGNet, GoogLeNet [10], and Inception [11]. However, deep network structures generate a large number of parameters, resulting in model redundancy. DenseNet [12] used a dense connection layer, and each layer can obtain the connected feature map of the previous layer. Model redundancy is reduced by feature reuse at each level of the network. The representative VGG-16 network [13] explored the relationship between the depth of a convolutional neural network and its performance by repeatedly stacking 3 × 3 convolution kernels and 2 × 2 max pooling layers. The convolution concatenation in VGG-16 network has fewer parameters than using a larger convolution kernel alone and has more nonlinear transformations than a single convolution layer. VGG-16 obtained more image features with a simple network structure, smaller convolutional kernels, and pooling layers, while avoiding excessive computational effort and overly complex structures.

### 2.2. Multireceptive Field-Based Skin Lesion Classification

High-level features are used to measure semantic similarity, and low-level features (edges and contours of pathological regions) can reflect image content. How to efficiently integrate the two features is the key to improving the classification model [14]. Szegedy et al. [15] combined the CNN features of different high and low layers applied to skin lesion classification. The different convolution layers would learn different weights according to different receptive fields, which can make the network explore more comprehensive pathological regions. Inception module of GoogLeNet [16] used multiple convolution layers with different kernels which are sampled at the same center to construct its receptive field module. Multireceptive fields are used to focus on different spatial positions of the object and its adjacent background, which is helpful to obtain high-quality features and enhance the distinguishability of features.

### 2.3. Recent Deep Learning-Based Skin Lesion Classification

Recent skin lesion classification studies have achieved recent performance [17, 18]. Gessert et al. [19] pretrained three neural network models and validated multiple balancing methods. They used metalearning methods for prediction and obtained 85.1% classification accuracy on ISIC2018 dataset. Shahin et al. [20] used an ensemble method to classify seven skin lesions by combining two network models, ResNet-50 and Inception-v3, and verified that the classification accuracy is as high as 89.9%. Amirreza et al. [21] studied the effect of image size on skin lesion classification based on pretrained CNN and transfer learning. On the ISIC2018 classification challenge testing set, the author's multiscale and multinetwork method yielded a balanced multiclass accuracy of 86.2%. Al-Masni et al. [22] segmented the pathological regions and then applied multiple convolutional network classifiers for lesion classification. The authors chose ResNet-50 by testing a number of established neural networks, which demonstrated its excellent performance with a classification accuracy of 89.28%. Zillur et al. [23] proposed a weighted average ensemble learning-based model to classify skin lesions, using five deep neural network models (ResNeXt, SeResNeXt, ResNet, Xception, and DenseNet) to find the best combination in the ensemble. Finally, an average classification accuracy of 88% was obtained. Abayomi-A et al. [24] created synthetic melanoma images by oversampling data in a nonlinear lower-dimensional embedding manifold. The augmented images were used to train the Squeeze Net deep learning model. Nawaz et al. [25] proposed a UNET model based on DenseNet77. The authors introduced the DenseNet77 network at the encoder unit of the UNET method to compute a more representative set of image features. The key points of the computation are subsequently segmented by the decoder of the UNET model.

In summary, a single network needs to be designed with a deeper network structure to learn as many pathological features as possible, but widening the depth and width of the network does not improve the classification performance of the model [26, 27]. Therefore, we employ a two-stream network to compensate for the shortcomings of a single network. Meanwhile, VGG-16 in our two-stream network fuses the residual structure, broadening the network depth while also focusing on more pathological regions. Thus, we decided to use DenseNet and VGG-16 to design our two-stream network model to obtain finer-grained pathological features and improve the classification accuracy of skin lesions.

## 3. Materials and Methods

### 3.1. Dataset

**Figure 2:**
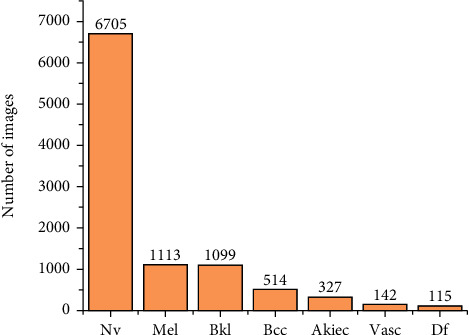
Distribution of lesion types in the dataset.

ISIC means International Skin Imaging Collaboration. It is the largest and public dataset of skin images and for medical image classification. In our model, the published ISIC2018 Task 3 dermoscopic image dataset [28] is used (also called the Human Against Machine (HAM) 10000 dataset) [29], which consists of 10,015 dermoscopic images, and each of which is a 600 × 450 three-channel RGB image. The dataset includes seven skin lesions, namely, actinic keratosis and intraepithelial carcinoma (Akiec), basal cell carcinoma (Bcc), benign lesions of the keratosis (Bkl), dermatofibroma (Df), melanoma (Mel), melanocytic nevus (Nv), and vascular disease (Vasc). The seven skin lesion types are shown in Figure 1. As can be seen from Figure 1, the high interclass similarity makes it difficult to classify lesions by the naked eye. Moreover, many images include hairs that are present on the skin, which significantly reduces the classification ability of the model. Although the ISIC2018 dataset provides 10,015 images as the training set and ground truth category labels with annotations, it is far from the amount of data needed for our model in the training and evaluation process. Thus, the dataset still needs to be manually divided. The component distribution of each lesion in the dataset is shown in Figure 2. The maximum and minimum numbers of images for different classes are 6705 and 115, respectively. Therefore, we perform a dataset preprocessing operation before the images are sent to the network.

### 3.2. Skin Lesion Multiclassification Model

To better improve the classification accuracy of skin lesions, we design a multiscale feature fusion model. Our model structure is based on DenseNet-121 network and improved VGG-16 network. We concatenate the output features of the two networks and use the multireceptive field to obtain multigranularity and multiscale global features. The feature fusion module is to enhance the ability to distinguish the pathological regions and background regions.

**Figure 3:**
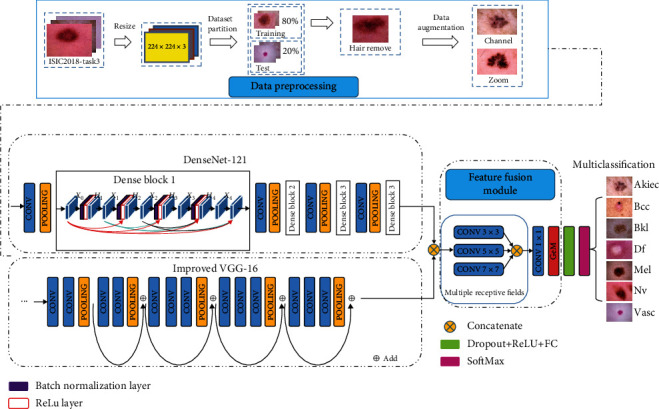
The framework of our model. Our model consists of four stages: data preprocessing, two-stream network, feature fusion module, and multiclassification.

Specifically, our network framework consists of preprocessing dataset, two-stream network, feature fusion module, and multiclassification. In the preprocessing step, we dehair the skin lesion images and use six data augmentation operations to improve the generalization ability of the network. To obtain and exploit more pathological features, we use two networks in parallel in the two-stream network. The two-stream networks are DenseNet-121 and improved VGG-16, respectively. We add a residual structure to the original VGG-16 to deepen the depth of the network without increasing the parameters. We use a VGG-16 network with residual structure to remove redundant information from correlations between different feature sets and further obtain contextual correlations in pathological regions. In the feature fusion module, we obtain multiscale regions by adding multireceptive field. Moreover, we abandon the traditional pooling operation and use the GeM pooling operation to balance the compression of the features, which can improve the classification accuracy. We use the softmax function as a classifier for multiclassification. Overall, the framework of our model is shown in Figure 3.

The steps of our two-stream network for skin lesion classification are as follows:

1. The skin lesion images are resized to 224 × 224 × 3 and divided into training set, test set, and validation set in the ratio of 7 : 2 : 1
2. The hair removal algorithm and six data enhancement operations are performed on the training set to reduce noise interference and balance the number of seven types of skin lesion images after preprocessing
3. The improved two-stream network model based on multireceptive fields is built to obtain more pathological features to improve the model classification performance
4. The seven classifications of skin lesion images are achieved using softmax classifier

#### 3.2.1. Data Preprocessing

**Figure 4:**
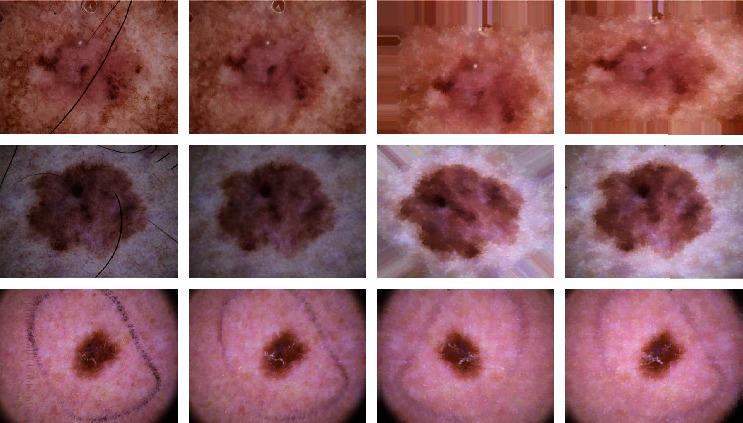
Examples of three groups of skin lesions by preprocessing. The first column shows the three sets of original images. The second column shows the corresponding dehaired images, and the third and fourth columns show the augmented images. We can observe that the hair in the pathological region is well removed.

In this subsection, we describe the preprocessing of the dataset in detail, including resizing, hair removal, and data augmentation, as can be seen in Figure 4.

- Resize: the ISIC2018 dataset contains high-resolution images. The resolution of all skin lesion images is 600 × 450 pixels, which requires high computational cost if used directly for training. Therefore, we resize all images from 600 × 450 to 224 × 224 as required by the model. Based on the literature [30], we select 70% of the data as the basic training set, 20% of the data as the basic test set, and the remainder of the data as the basic verification set, and the number of each lesion in the training set, test set, and verification sets is divided into a ratio of 7 : 2 : 1. The sample distribution after dataset division is shown in Table 1
- Hair removal [31]: the ISIC2018 datasets are often characterized by hair-like regions within the skin lesions, which would interfere with the model's extraction of pathological features. Thus, we dehair all images to reduce the hair interfere. We convert the original image to a grayscale image, detect the hair contour using the black-hat operation, and create the mask. We apply an image inpainting technique based on the fast marching method (FMM) [32] to remove the mask containing only the hair from the original image and repair the void pixels. Hair removal operation can remove hair in pathological regions very well. Although some pixel information is lost, the removed pixels are mostly hair pixels and do not have a large impact on the feature extraction of the model
- Data augmentation: although the ISIC2018 dataset contains 10015 images, it cannot meet the number of large-scale data required for deep learning methods. Thus, we perform six data augmentation operations including randomly rotating, horizontal and vertical shifting, random zooming, random transforming, flipping, and resizing on hair removal training samples. We expanded the samples of the other six skin lesions in the training set except for melanocytic nevus (Nv) samples to enlarge the sample data. Thus, the other number of samples is approximately equal to the number of melanocytic nevus (Nv) samples, which avoid the overfitting caused by too few samples. The sample distribution of the training set after data augmentation is shown in Table 1

#### 3.2.2. Our Two-Stream Network Structure

We chose a deep network DenseNet-121 and a shallow network VGG-16 to construct our two-stream network. To increase the network depth and exploit more pathological features without adding extra parameters, we improve the VGG-16 by adding the residual structure. We use two networks to better distinguish pathological regions from background regions and improve the classification accuracy of skin lesions.

DenseNet-121 is a tightly connected CNN with well resistance to overfitting. The complexity of the network increases with depth (combination of more nonlinear functions), but DenseNet-121 has a direct connection between any two layers. Therefore, it can make full use of previous features, and it is easier to obtain richer pathological information. Compared with the sparsity of VGG-16, the DenseNet-121 is more compact, and the feature generated by the DenseNet-121 is more powerful, which makes up for the deficiency of VGG-16. Moreover, the compact structure can effectively reduce the gradient disappearance and improve the efficiency of feature utilization and enable the model to pay attention to the pathological information of a larger region.  Table 2 is the network structure and output size of DenseNet-121.

**Figure 5:**
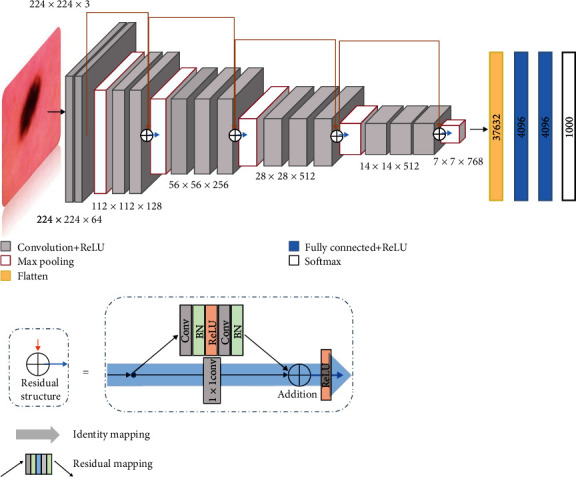
VGG-16 fusion residual structure network model.

Since the densely connected layer repeatedly utilizes features from the previous layer, the correlation between features affects the model classification performance. To compensate for the redundancy caused by feature reuse and enhance the transfer of pathological information between layers in the VGG block, we use VGG-16 fusion residual network for feature fusion to eliminate redundancy. The original VGG-16 network structure is shown in Table 3 and mainly includes 5 VGG blocks. Each VGG block contains 2 or 3 convolution layers and a max pooling layer. It can be observed that the VGG-16 network structure is simple without redundant layers to interfere with the model classification performance. For skin lesion images, we must explore fine-grained local features to ensure accurate classification, especially contour descriptors with lesion tendency. We add residual structures before and after each block of the VGG-16 network to extract multiscale features. The residual structure is divided into two parts: identity mapping and residual mapping. Identity mapping occurs when the output features of the previous layer are directly input to the next layer, and we use a 1 × 1 convolutional layer to match the number of feature channels. Residual mapping occurs when the input features of the previous layer are input to the next layer through the superposition of nonlinear changes. We use a unit addition operation on the residual structure output features to obtain more pathological features, which does not add additional trainable parameters. Moreover, we use a residual structure to obtain the feature information of the upper layer input to reduce the feature loss between different blocks. The fuse of the residual structure [8] simplifies the training of the network while preserving the information integrity. The residual structure increases the ability of gradient cross-layer propagation and further improves the classification accuracy of our model. The model structure of the VGG-16 fusion residual network is shown in Figure 5.

Finally, we remove the FC layer from both DenseNet-121 and improved VGG-16. This keeps the consistent dimensions of the output feature maps, so that the feature fusion module can be performed on the output feature maps.

#### 3.2.3. Feature Fusion Module

Due to the uneven distribution of pathological regions in the skin lesion images with different sizes and poor continuity, we determine the distribution of the extracted features by the size of the convolution kernel. The use of smaller convolutional kernels is biased towards extracting more local feature information, while the use of larger convolutional kernels is biased towards extracting more global pathology image features. However, to ensure that the output feature mapping is large enough, if we only use smaller convolutional kernels, a deeper network is often required, which will easily lead to network overfitting. The use of larger convolutional kernels will ignore local information, and the stacking of larger convolutional kernels will increase the computational effort and lead to a decrease in model efficiency.

To obtain information on a larger range of skin lesions, we have designed the feature fusion module to obtain the multireceptive field of skin lesions. As shown in the feature fusion module in Figure 3, the multireceptive fields are composed of multiple convolution layers with different kernel sizes, including 3 × 3 convolutional layers, 5 × 5 convolutional layers, and 7 × 7 convolutional layers. The role of multireceptive field is to cover a larger region of skin lesions for obtaining more pathological regions. Different convolutional layers will learn different weights according to different receptive fields, and the smaller convolution kernel and the larger convolution kernel complement each other. They explore a more comprehensive pathological region, which helps to improve the overall accuracy of the model. We concatenate the feature maps of all convolutions with different receptive fields and enter them into the 1 × 1 convolutional layer+ReLU layer to perform channel-integrated and nonlinear processing.

To weigh the pathological regions obtained by the features, we use a GeM pooling operation [33, 34] in the feature fusion module. We take a 1 × 1 × 1536 feature vector *X* as the input and a vector *H* as the output of the pooling process. In the case of using max pooling, this vector *H*_*n*_^(*m*)^ is given by

(1) $$\begin{array}{c} H^{\left(m\right)}={\left[H_{1}^{\left(m\right)}\cdots H_{n}^{\left(m\right)}\cdots H_{K}^{\left(m\right)}\right]}^{T},\;H_{n}^{\left(m\right)}=\underset{x\in X_{k}}{\max}x, \end{array}$$

where *K* is the number of channels of the feature map. Let *X*_*k*_ be the set for feature map *n* ∈ (1, *K*). The network output consists of *K* such feature maps. The *m* refers to the max pooling operation and the *x* is all the features obtained in *X*_*k*_.

And in the case of using average pooling, this vector *H*_*n*_^(*a*)^ is given by

(2) $$\begin{array}{c} H^{\left(a\right)}={\left[H_{1}^{\left(a\right)}\cdots H_{n}^{\left(a\right)}\cdots H_{K}^{\left(a\right)}\right]}^{T},\;H_{n}^{\left(a\right)}=\frac{1}{\left|X_{k}\right|}\underset{x\in X_{k}}{\sum }x. \end{array}$$

Instead, we exploit the GeM pooling, and this vector *H*_*n*_^(*g*)^ is given by

(3) $$\begin{array}{c} H^{\left(g\right)}={\left[H_{1}^{\left(g\right)}\cdots H_{n}^{\left(g\right)}\cdots H_{K}^{\left(g\right)}\right]}^{T},\;H_{n}^{\left(g\right)}={\left(\frac{1}{\left|X_{k}\right|}\underset{x\in X_{k}}{\sum }x^{P_{k}}\right)}^{1/P_{k}}. \end{array}$$

*P*_*k*_ is a hyperparameter that adjusts the weights of the two pooling operations. Max pooling and average pooling are special cases of GeM pooling. When *P*_*k*_⟶∞, it is max pooling and average pooling for *P*_*k*_⟶1. The *H*^(*g*)^ ultimately consists of the values of each feature map after GeM pooling, and its dimensionality is equal to *K*. We have trained and tested different *P*_*k*_ values and selected appropriate parameters to get better classification results in the following comparative experiments.

In the multiclassification module, we use the softmax classifier to achieve multiclassification of skin lesions.

## 4. Experiments and Results

In this section, we first introduce our experimental environment and the evaluation metrics. Then, we present the results of our ablation experiments to evaluate the performance of the network model. Next, we compare the performance of our model with other models. Finally, we introduce the skin lesion classification system, which can be used to assist doctors in diagnosing.

We use the deep learning framework TensorFlow [35] to build the network model. We use Python 3.8 as the programming language on the Windows 10 operating system with an Intel i7-10700F CPU and NVIDIA GeForce GTX2060 GPU.

### 4.1. Evaluation Metrics

The evaluation metrics include precision, recall, *F*1-score, and accuracy. Precision in Equation (4) is used to measure the prediction accuracy of the classifier for a certain category. Recall in Equation (5) is used to measure the coverage of the classifier's prediction results for a certain category. *F*1-score in Equation (6) is used to measure the accuracy and coverage of the classifier's prediction for a certain category. Accuracy in Equation (7) is the average accuracy that is used to calculate the overall, where TP, TN, FP, and FN represent the true positive, true negative, false positive, and false negative, respectively. We also use the receiver operating characteristic (ROC) curve and area under the curve (AUC) as the evaluation criteria of model performance [36]. The ROC and AUC can well describe the classification performance of the classifier for samples with uneven distribution. To extend our metrics to multiclassification, the macro- and microaverages are also calculated [23]. The macro- and microaverages mean that when calculating multiclass indicators, different weight methods are used to assign all samples.

(4) $$\begin{array}{c} \text{Precision}=\frac{\text{TP}}{\text{TP}+\text{FP}}, \end{array}$$

(5) $$\begin{array}{c} \text{Recall}=\frac{\text{TP}}{\text{TP}+\text{FN}}, \end{array}$$

(6) $$\begin{array}{c} F1-\text{score}=\frac{2\text{ Precision}\times \text{Recall}}{\text{Precision}+\text{Recall}}, \end{array}$$

(7) $$\begin{array}{c} \text{Accuracy}=\frac{\text{TP}+\text{TN}}{\text{TP}+\text{TN}+\text{FP}+\text{FN}}. \end{array}$$

### 4.2. Ablation Experiment

We conducted three sets of ablation experiments in Section 4.2, and the training set is included by hair removal and data augmentation. The distribution of the training and test sets listed is in Table 1.

#### 4.2.1. The Performance of a Two-Stream Network

We compare our two-stream network model with the VGG-16, improved VGG-16, and DenseNet-121, respectively. The experimental results are shown in Table 4. Compared with VGG-16, our improved VGG-16 has better classification performance, with precision, recall, *F*1-score, and accuracy increase of 11.42%, 10.43%, 11%, and 15.12%, respectively. It can be seen from Table 3 that the main layers in the VGG-16 network are only the convolutional layer and max pooling layer, which causes the VGG-16 network to ignore the correlation between different blocks. And the VGG module has poor ability to aggregate feature information due to its simple stacking. The residual structure strengthens the correlation between blocks. It well solves the gradient degradation due to feature redundancy as the depth of the network increases.

Compared with the classical networks, our two-stream network has better classification performance; the precision, recall, *F*1-score, and accuracy are 83.53%, 95.04%, 88.91%, and 91.24%, respectively. Our two-stream network is a sparse-compact network structure that can complement the strengths of both networks, where the DenseNet-121 obtains more detailed pathological features due to its compact network structure and the improved VGG-16 can well capture the local features of pathological regions due to the added residual structure. This shows that our two-stream network can effectively obtain more fine-grained information in the skin lesion images, making the feature representation more robust and improving the classification accuracy of the model.

#### 4.2.2. The Performance of Multireceptive Field

To better illustrate the effectiveness of multireceptive field, we compare the improved VGG-16 and our model that both removed the multireceptive field. The results are shown in Table 5, where (-) stands for removing the multireceptive field module. Compared with the model without multireceptive field, our model has better classification performance, with precision, recall, *F*1-score, and accuracy increase of 0.65%, 0.94%, 0.78%, and 1.07%, respectively. In the absence of multireceptive field, the network is unable to distinguish the boundaries of pathological regions and obtain more local information. We can obtain a wider range of local information by using multireceptive field. Avoiding the loss of image detail texture by too small or too large receptive fields can effectively enhance the discriminability of pathological features and improve the accuracy of model classification.

#### 4.2.3. The Performance of Different *P*_*k*_

According to literature [33], we debug the *P*_*k*_ value in the GeM pooling operation ranging from 1 to ∞. The accuracy of the model drops when *P*_*K*_ is 5, at which point we stop testing. The GeM pooling operation is a weighting of the max pooling and average pooling to highlight the advantages of both pooling operations. As can be seen from Table 6, when the parameter *P*_*k*_ is 4, the classification accuracy of our model is the highest, reaching 91.24. So we set the value of *P*_*K*_ to 4. The GeM pooling operation we used is better than the classification accuracy obtained by the max pooling (*P*_*K*_ = 1) and average pooling (*P*_*K*_ = ∞) operations by 3.09% and 2.21%, respectively.

### 4.3. Visualization Results

**Figure 6:**
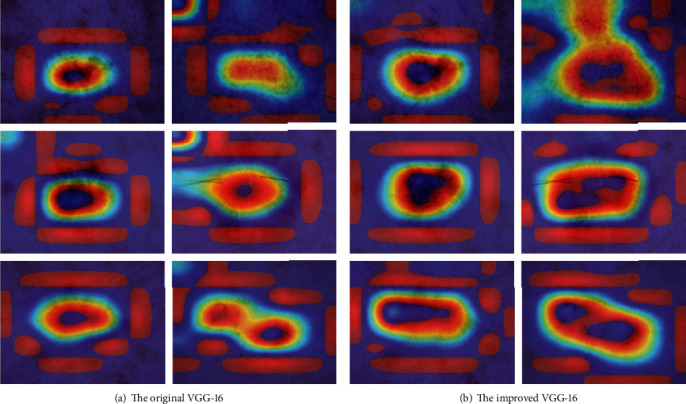
Visualization comparison between the original VGG-16 and the improved VGG-16.

Grad-CAM [37] is used to display the visualization results of the output feature map, as shown in Figure 6. We visualized some of the random images of the training set for judgment. To test whether the proposed network model can focus on the location of the center of the pathology, we visualized some of the random images of the training set.  Figure 6(a) shows the visualization results of the original VGG-16, and it is observed that the network only pays attention to two small parts of the pathological region.  Figure 6(b) shows the visualization results of the improved VGG-16, and it is observed that the network focuses on larger and broader pathological regions. The improved VGG-16 enables to pay more attention to the semantically meaningful parts of the lesions and enhance the ability of the network to learn discriminative representations. The results show that combining the residual structure can make the model better detect the lesion region. It can be seen that the improved VGG-16 network is more accurate for the feature extraction of pathological regions.

### 4.4. Training and Testing Results of Our Model Performance

#### 4.4.1. Model Loss

**Figure 7:**
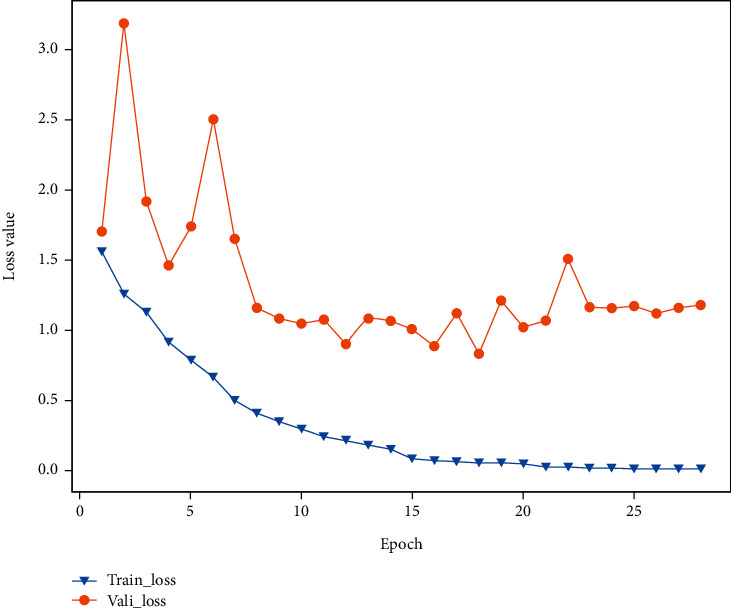
Line chart of training loss and validation loss.

Figure 7 shows the loss between the training and validation processes of our model. The *x*-axis represents the epoch, and the *y*-axis represents the loss. We choose a batch size of 16 and a learning rate of 0.0001 initially, which decreases to 1/10 of the original learning rate after a period of training. It is observed that the model is run 28 epochs with GPU acceleration. In the trials for the training model, if the epoch does not change for five consecutive times, the training will be stopped. Considering that increasing the number of epochs does not increase the performance while extending the training period, it is deemed appropriate to limit the training process to 28 epochs. As can be seen from Figure 7, the training loss of our model reaches a very low value, which indicates that our model has been effectively trained.

#### 4.4.2. Confusion Matrix

**Figure 8:**
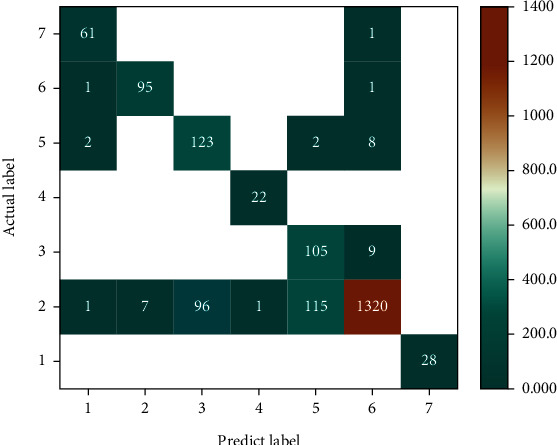
The confusion matrix of the model.

As a result of testing the model, the confusion matrix of seven different classes is shown in Figure 8. The *x*-axis represents the predicted skin lesion label (classes 0 to 6 represent Akiec, Bcc, Bkl, Df, Mel, Nv, and Vasc, respectively), and the *y*-axis represents the actual skin lesion label. The darker the color in the table, the greater the indicators of representative classification. It can be seen that there are a total of 222 Mel images in the testing set, and 115 of these images are misclassified as Nv, because the pathological region of the Mel image in the early stage is similar to Nv images. Therefore, it was misclassified the most, with an accuracy rate of only 47%. The classification accuracy of Vasc is 100% because of the significant difference in texture and color. It can be seen from the confusion matrix that our model has obtained a satisfactory result.

#### 4.4.3. Area under the ROC Curve

**Figure 9:**
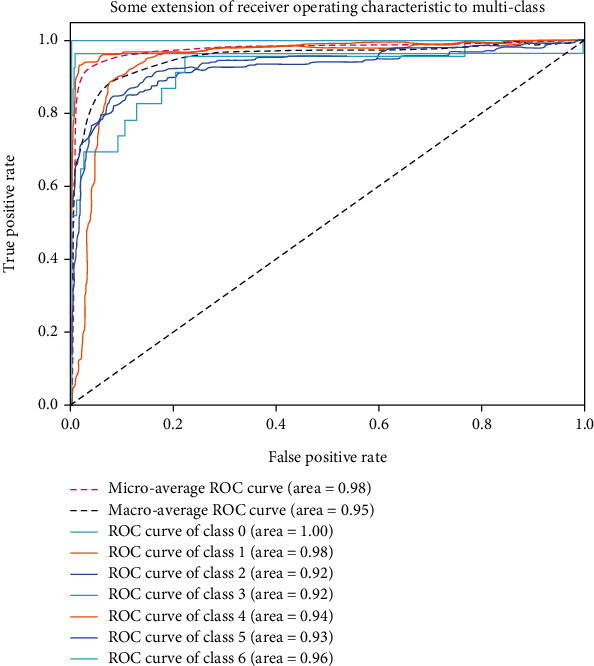
Area under the ROC curve. Note that classes 0 to 6 represent Akiec, Bcc, Bkl, Df, Mel, Nv, and Vasc, respectively.

To evaluate our classification model holistically across all classes, we also use macroaverages and microaverages. Because the ISIC2018 dataset has an unbalanced distribution. The macroaverage takes into account the distinction between classes, so we use this parameter to provide a good measure of the performance of our model on the studied dataset. The area under the ROC curve of our model is shown in Figure 9. AUCs of seven skin lesions are 1, 0.98, 0.92, 0.92, 0.94, 0.93, and 0.96, respectively. The microaverage of the ROC curve is 0.98, whereas its macroaverage is 0.95. This demonstrates that the model has excellent ROC curve scores for all classes. Therefore, our model has good classification performance in the multiclassification task of skin lesions.

### 4.5. Comparing to Other Existing Methods

To allow a fair comparison with previous works, all methods listed in Table 7 use the ISIC2018 dataset as the training set. With the exception of some Akiec images, the ISIC2018 dataset contains most of the HAM dataset images. Therefore, the HAM dataset can also be used to compare. Literature [19–21] all used more than three network models for feature fusion, resulting in too many parameters and redundant features in the models. Literature [22] applied convolutional neural network classifiers (i.e., Inception-v3, ResNet-50, Inception-ResNet-v2, and DenseNet-201) to segmented skin lesions for classification. Nevertheless, the authors fused the residual structure into Inception-v2 without better performance than the original ResNet-50 classification. Then, literature [23] proposed a weighted average ensemble learning-based model. They used five deep neural network models, namely, ResNeXt, SeResNeXt, ResNet, Xception, and DenseNet, as the base of the ensemble where the best weight combination was found by the grid search method. However, the authors did not consider the correlation between features. Literature [38] improved the data augmentation method and loss function and used the RegNetY-3.2GF-Drop model with medium complexity to achieve 86.4% classification accuracy. From the network improvement side, our two-stream network is a sparse and compact network structure. And the robustness of the final features can be improved by fusing the two networks. By adding the residual structures in VGG-16 and using multireceptive field, more pathological features are obtained for our model. Meanwhile, we fuse the output features of the two networks and appropriately remove the fully connected layer of the network, resulting in a significant reduction in the model parameters as well. We only use three different sets of convolution kernels to obtain multigranularity pathological features. In addition, our model obtains the complementary pathological region features as much as possible through the mechanism of multireceptive field. From the data processing side, our model simplifies the preprocessing process and further expands the data in terms of data augmentation. We have enough data to reduce the overfitting of the model. In summary, the classification accuracy of our model is 91.24%, and the macroaverage rate is 95%, which is better than the above methods.

### 4.6. Skin Lesion Classification System Based on Two-Stream Model

We have designed a skin lesion classification system based on our two-stream model. The framework is built using the Python Flask [39]. Our web system has a simple interface for experts or patients to easily use. For classification, specific test images can be uploaded to the system (select images). Once the test images are classified, the results and time consumed will be displayed on the page.

**Figure 10:**
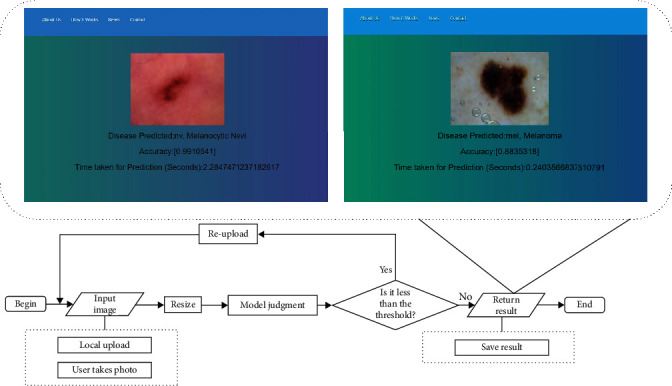
Skin lesion classification system.

The implementation process of the system is shown in Figure 10. Firstly, we input a skin lesion image, and we require the upload format to be RGB image. For the convenience of users, we provide two types of uploads: photo and local upload. Users can take photos and upload images of any size. Secondly, the system uniformly adjusts the image to 224 × 224 × 3. Thirdly, the image is sent to our two-stream network model to determine what kind of disease is being classified. Since the model uses the softmax classifier as the classification layer, this layer will output the probability of skin lesions of each category. The system will return the label corresponding to the maximum probability value and time consumed as the result to the user. Fourthly, the system determines whether it is below the threshold value. Here, to classify the malignant disease, melanoma, we set the threshold to 0.9. When the probability value of the largest category label is less than 0.9, the user is requested to reupload the images. Conversely, if the threshold is higher than 0.9, the result display is returned, showing the probability of the disease category at the highest threshold and the time required for analysis. And the classification results can be saved directly for easy viewing by users. The following is the system execution flow:

1. Input RGB image (you can upload locally or take photos)
2. Resize the image
3. Send to two-stream network model for classification
4. Judgment: if the threshold value is less than 0.9, return to the first step; otherwise, proceed to the fifth step
5. Return the results and save the analysis results
6. End

The test of the skin lesion classification system was carried out as follows. The skin lesion images uploaded by the user are first classified according to our two-stream model. Then, the accuracy is confirmed by comparing the dataset stored on the server, resulting in the lesion type with the highest probability and consumed time. We engineered tests by using uploaded data on different seven types of skin lesions. According to the test results in Table 8, the model correctly classified 548 of the 600 images. The classification system was able to accomplish a classification speed of less than 1 second. We tested from our own collection and operation of the skin dataset, and these results show the accurate classification performance of our system. We emphasize that our model can provide accurate diagnostic information for experts or patients.

From Table 8, we find that the test success rate of the model is 91%, which is roughly in line with the training accuracy of the model. The most misclassified lesion type observed in the test was Df, and it was also noted that Bkl and Nv were confused. The possible reason is that the color of the lesion region of Df and Bkl is similar to the skin color, making it difficult to classify correctly. As Nv is a common benign nevus with different color and shapes of growths, it is the most numerous in the dataset and the most easily confused with other skin lesions. In the ISIC2018 dataset, the pathological symptoms of Nv are similar to other categories, and Nv itself is asymmetric, irregular, and rough. Therefore, the classification success rate of Nv is also low. In addition, the classification success rate was lower in the presence of hair and darker skin color around the lesion, so contrast enhancement of the image and hair removal would be beneficial. In future work, increasing the number of taken dermatological images in the test will also provide more generalized results.

Effective classification of skin lesions can allow patients to go to the hospital in time to improve the possibility of survival. In summary, our model has good performance on the skin lesion multiclassification task.

## 5. Discussion

The main contribution of this work is designing a two-stream network for multiscale feature fusion for skin lesion classification, which achieves good performance on the highly unbalanced seven-class dataset ISIC2018. The performance of our model in precision, recall, accuracy, and macroaverage is 83.53%, 95.04%, 91.24%, and 98%, respectively. We use the improved VGG-16 structure by using residual structures. Residual structures are fused before and after each layer. Thus, the features of the previous layer can be transported to the next layer through identity mapping and residual mapping. Another network model DenseNet-121 enhances the propagation of features by designing densely connected dense blocks, reduces the number of parameters of the network, and alleviates the gradient dispersion problem caused by the overly deep network model, thus improving the classification rate of the deep neural network. In the future, we will use different data augmentation methods to train and combine more models to better achieve the classification performance to meet the medical diagnosis needs. The two-stream network we chose is the DenseNet-121 and improved VGG-16. The model is not a lightweight network, and we will use lightweight network to design the experiment later, which is a limitation of our method. And we only did the experiments on ISIC2018; later, we will choose more ISIC datasets and experiments using real data in the clinic.

## 6. Conclusions

Malignant skin lesions have a high mortality rate and have high interclass similarity and intraclass variations. Therefore, a reliable classification system would be of great help to clinicians in the early detection of malignant skin lesions. In this paper, we propose a multiscale feature fusion model for skin lesion classification. We use DenseNet-121 and an improved VGG-16 network as our two-stream network to complement the advantages of a single network. Noteworthily, we fuse the residual structure of the original VGG-16 model to optimize the model without adding parameters. Then, we exploit the feature fusion module to obtain multiscale pathological information. In summary, our model achieves 91.24% test accuracy and 95% macroaverages on the ISIC2018 dataset. Finally, we design a skin lesion classification system in our two-stream network to help physicians effectively classify a patient's early-stage skin lesions.

Therefore, improving the ability to automatically classify based on skin lesion images is necessary to help physicians classify skin lesions and assist in early medical diagnosis. In the future, we will try to design a complete auxiliary diagnosis system based on our model.

## Figures and Tables

**Table 1:** The sample distribution after dataset division and the training set after data augmentation.

Category	Basic training set	Augmentation training set	Basic test set	Basic verification set
Nv	4697	4697	1339	669
Mel	780	5439	222	111
Bkl	771	5383	219	109
Bcc	361	5040	102	51
Akiec	230	4251	65	32
Vasc	100	5600	28	14
Df	81	4536	23	11
Total	7020	34946	1998	997

**Table 2:** Network structure and output size of DenseNet-121.

Layers	Output size	DenseNet-121
Convolution	112 × 112 × 64	

Pooling	56 × 56 × 64	

Dense block (1)	56 × 56 × 256	(1 × 1 conv) × 6, (3 × 3 conv) × 6

Transition layer (1)	56 × 56 × 128	
	28 × 28 × 128	

Dense block (2)	28 × 28 × 512	(1 × 1 conv) × 12, (3 × 3 conv) × 12

Transition layer (2)	28 × 28 × 256	
	14 × 14 × 256	

Dense block (3)	14 × 14 × 1024	(1 × 1 conv) × 24, (3 × 3 conv) × 24

Transition layer (3)	14 × 14 × 512	
	7 × 7 × 512	

Dense block (4)	7 × 7 × 768	(1 × 1 conv) × 16, (3 × 3 conv) × 16

**Table 3:** Network structure and output size of VGG-16.

Layers	Output size	VGG-16
VGG block (1)	224 × 224 × 64	(3 × 3 conv) × 2

Pooling	112 × 112 × 128	

VGG block (2)	112 × 112 × 128	(3 × 3 conv) × 2

Pooling	56 × 56 × 256	

VGG block (3)	56 × 56 × 256	(3 × 3 conv) × 3

Pooling	28 × 28 × 512	

VGG block (4)	28 × 28 × 512	(3 × 3 conv) × 3
	28 × 28 × 1024	
	28 × 28 × 1536	

Pooling	14 × 14 × 768	

VGG block (5)	14 × 14 × 768	(3 × 3 conv) × 3
	14 × 14 × 512	
	14 × 14 × 768	

Pooling	7 × 7 × 768	

**Table 4:** Our model performance compared to classical networks.

Methods	Precision (%)	Recall (%)	*F*1-score (%)	Accuracy (%)
VGG-16	66.43	75.09	70.50	72.48
Improved VGG-16	77.85	85.52	81.50	87.60
DenseNet-121	80.94	87.61	84.14	88.14
Our model	83.53	95.04	88.91	91.24

**Table 5:** Our model performance compared to multireceptive field.

Methods	Precision (%)	Recall (%)	*F*1-score (%)	Accuracy (%)
Improved VGG-16 (-)	76.47	83.23	79.71	86.32
Improved VGG-16	77.85	85.52	81.50	87.60
Our model (-)	82.88	94.10	88.13	90.17
Our model	83.53	95.04	88.91	91.24

**Table 6:** Our model accuracy compared to used different *P*_*K*_.

Parameter value of *P*_*K*_	1	2	3	4	5	∞
Accuracy	88.15	89.87	90.58	91.24	89.17	89.03

**Table 7:** Comparison with other existing methods.

Method	Date	Precision (%)	Recall (%)	Para (M)	Accuracy (%)	Macroaverage (%)
DenseNet, SENet, ResNeXt [19]	2018	—	—	—	85.10	—
InceptionV3+ResNet-50 [20]	2018	86.2	79.60	—	89.90	—
MSM-CNN [21]	2020	91.30	—	—	86.20	98
ResNet-50 [22]	2020	—	81.00	23.54	89.28	—
ResNeXt, SeResNeXt, ResNet, Xception, DenseNet [23]	2021	87.00	94.00	—	88.00	93
RegNetY-3.2GF-Drop [38]	2021	—	—	15.30	86.40	97
Our method		83.53	95.04	19.70	91.24	98

**Table 8:** Skin lesion classification system results.

Groups	Number of correct predictions	Number of wrong predictions	Success rate (%)	Most confused lesion
1	93	7	93	Df
2	89	11	89	Df, Bkl
3	91	9	91	Nv, Df
4	93	7	93	Df
5	89	11	89	Df, Bkl
6	93	7	93	Df
	548	52	91	

## Data Availability

The datasets generated or analysed during this study are available from the corresponding author on reasonable request.
